# African histoplasmosis of the penis

**DOI:** 10.1093/omcr/omaa043

**Published:** 2020-06-25

**Authors:** Tchin Darré, Matchonna Kpatcha, Toukilnan Djiwa, Edoé Sewa, Améyo Monique Dorkenoo, Mouhamed Kouyaté, Gado Napo-Koura

**Affiliations:** 1 Department of Pathology, University Teaching Hospital of Lomé, Lomé, Togo; 2 Departement of Urology, University Teaching Hospital of Lomé, Togo; 3 Department of Mycology, University Teaching Hospital of Lomé, Lomé, Togo; 4 Department of Pathology, University Teaching Hospital of Treichville, Abidjan, Ivory Coast

**Keywords:** Penis, African histoplasmosis, *Histoplasma duboisii*, Ulcer

## Abstract

African Histoplasmosis is deep mycosis caused by *Histoplasma duboisii* and genitourinary involvement is extremely rare. We report a case of African histoplasmosis in a 27-year-old subject with painful penis ulcer. Ulcer edge biopsy had revealed inflammatory granulomas made of epithelioid cells, lymphoplasmocytes, polynuclear eosinophils and giant multinucleated cells, with ovoid yeasts surrounded by a clear halo. PAS and Grocott stains revealed numerous fungal structures with a morphology measuring 7 to 15 nm. The diagnosis *Histoplasma capsulatum var. duboisii* was placed and the patient put on itraconazole (400 mg/day) for six months with a good course. African histoplasmosis of the subject penis is an extremely rare entity. The diagnosis of certainty often makes use of histology and mycological examination, and makes it possible to eliminate differential diagnoses such as cryptoccocosis, tuberculosis or cancer.

## INTRODUCTION

Histoplasmosis is a fungal infection caused by *Histoplasma capsulatum*, a dimorphic fungus with two distinct varieties affecting the humans: *Histoplasma capsulatum var capsulatum* and *Histoplasma capsulatum var. duboisii* [1]. The first variety is commonly found in the United States and Latin America with sporadic cases reported worldwide [1]. The second variety, presented as a skin infection, is limited to sub-Saharan Africa [2]. Clinically, African histoplasmosis is characterized by lesions whose location and course often suggest a tuberculous etiology [3]. The preferential locations of African histoplasmosis are osteoarticular, ganglionic and pulmonary [2]. Genitourinary skin damage occurs in 4 to 11% of patients and results from secondary skin invasion in patients with disseminated infection [4]. Primary genitourinary histoplasmosis is an extremely rare entity [4]. We report a case of *Histoplasma capsulatum var. duboisii* of the penis in a young Togolese 27 years old. This case underlines the rarity of the localization of affection to the penis and the difficulties of its management.

## CASE PRESENTATION

A 27 year old student, black, rural resident, had consulted in the urology department for chronic non-healing ulceration onset for 8 months associated with dysuria and burning urination two weeks ago. The beginning was marked by painless erythematous nodules measuring 1x1cm which gave rise to multiple ulcers on the glans of the penis and around the urethral meatus. There was no evidence of trauma, surgery, weight loss, fever, cough or other constitutional symptoms. He had been vaccinated with the BCG vaccine at birth. He had tried different herbal treatment modalities of unknown nature, without success. The examination noted a good general condition of the patient, and the conjunctivae were well colored.

**Figure 1:**
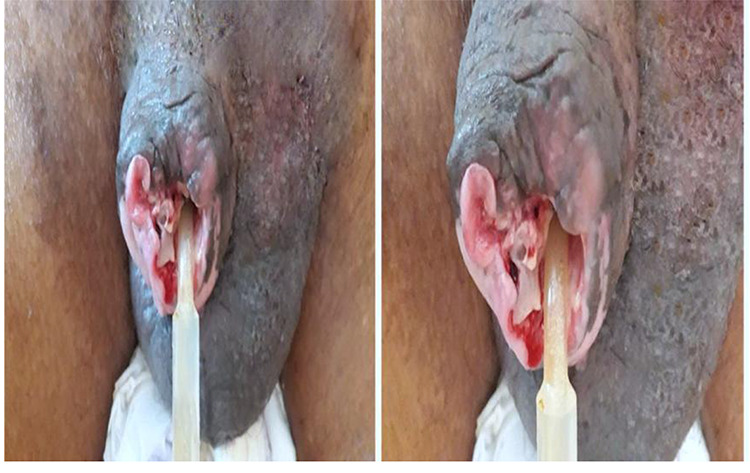
Macroscopy of a large gland ulcer with necrotic and haemorrhagic remanence.

Examination of the genitourinary organs shows a large ulcer with irregular contours, circumferential shape of 6x4x3cm of the glans. The ulceration fundus is dirty, hemorrhagic and necrotic, with a poorly limited urethral orifice (Fig. 1). The bilateral inguinal lymph nodes were enlarged between 0.5 and 2 cm in diameter, firm, and mobile. The rest of the clinical examination of the genitals was normal. Biological studies have revealed a high sedimentation rate (54 mm for the first hour). Blood sugar, serum urea and creatinine were normal. Serological tests for HIV and VDRL were negative.

**Figure 2:**
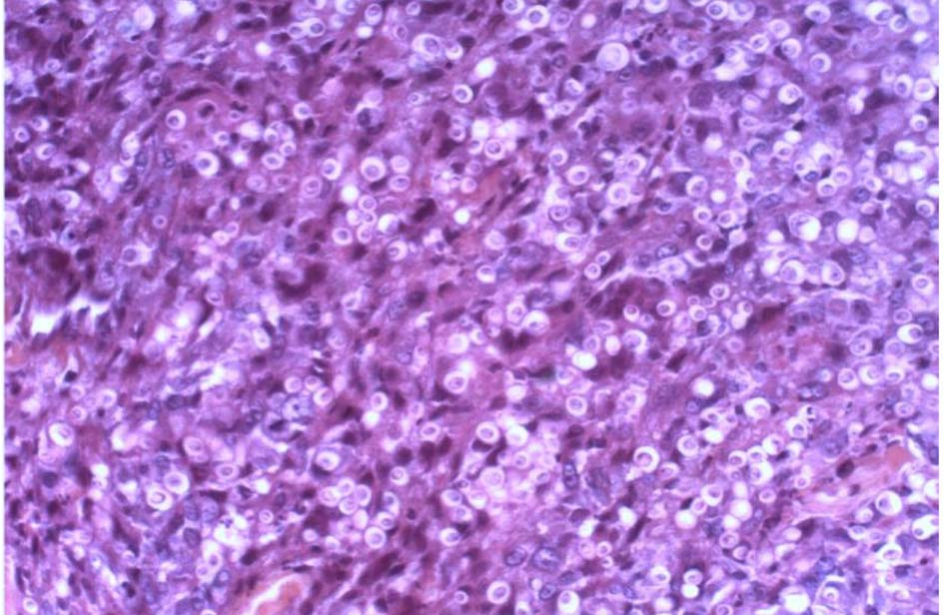
Photomicrograph showing large yeasts surrounded by a clear halo of *Histoplasma capsulatum var. duboisii* (H and E, X 400).

**Figure 3:**
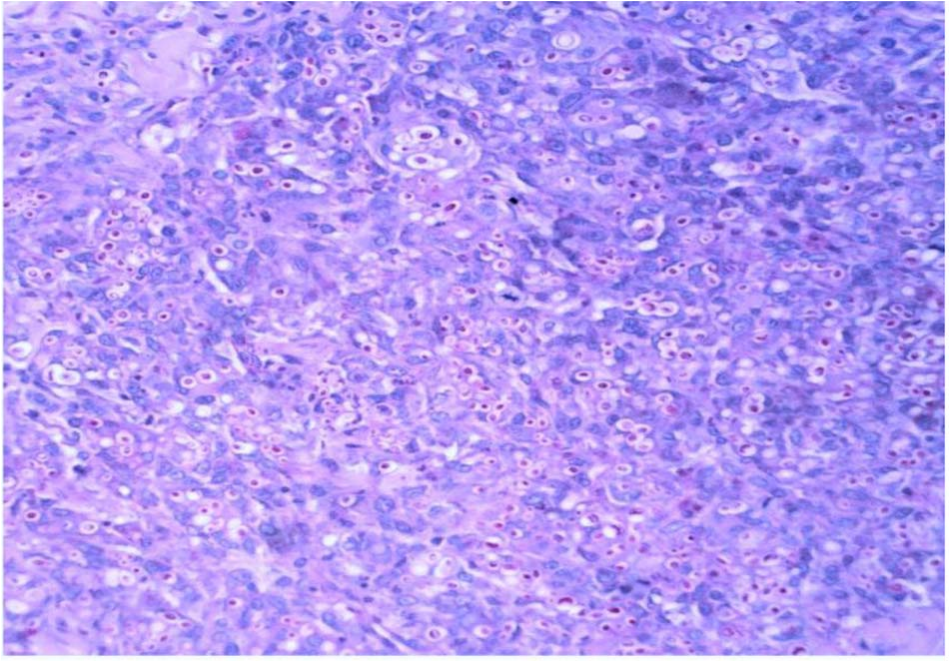
Photomicrograph showing *Histoplasma capsulatum var. duboisii* yeast colored in purple red (PAS, X 200).

**Figure 4:**
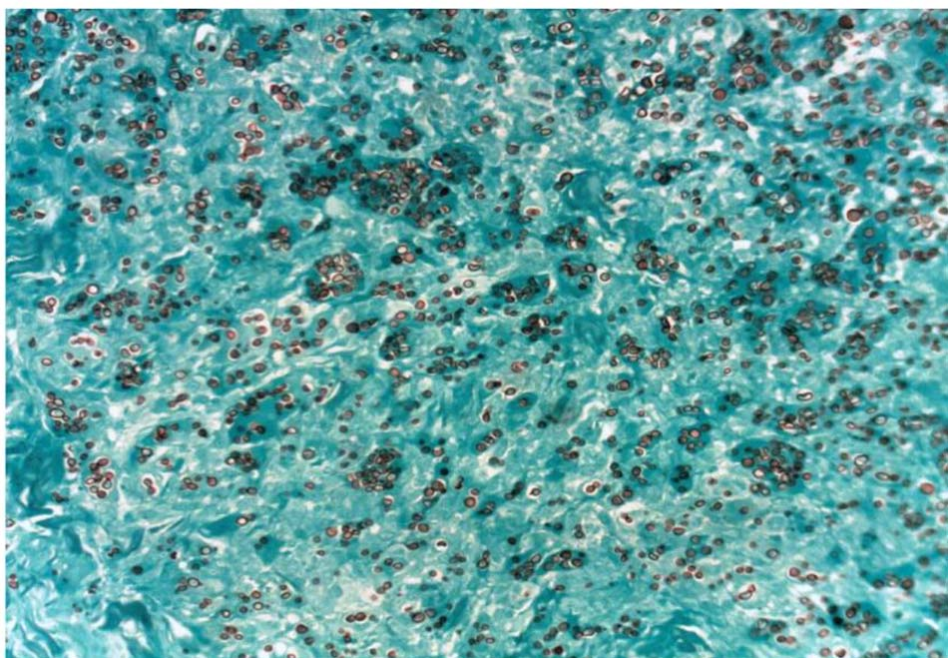
Photomicrograph showing *Histoplasma capsulatum var. duboisii* yeast well individualized (Gomori Grocott, X 200).

Microscopic examination of the urine and culture revealed no abnormalities. Bacilli were not observed either in the scraping of the base of the ulcer, urine or sputum. Ultrasound of the kidneys and bladder was within normal limits. The chest X-ray was also normal. A biopsy was made and anatomo-pathological examination carried out on the samples. The histopathological examination presented inflammatory granulomas made of epithelioid cells, lymphoplasmocytes, polynuclear eosinophils and giant multinucleated cells with ovoid yeasts surrounded by a clear halo (Fig. 2). The PAS and Grocott stains revealed numerous fungal structures with a morphology measuring 7 to 15 nm (Figs. 3 and 4). The diagnosis of histoplasmosis by *Hstoplasma capsulatum var. duboisii* has been confirmed. The patient was put on treatment with itraconazole (400 mg/day) for twelve months with a favorable evolution.

## DISCUSSION

### EPIDEMIOLOGY

Our case of African *histoplasmosis var. duboisii* was diagnosed in a young male subject. The localization of African Histoplasmosis in the penis is extremely rare, and only around twenty cases have been reported in the literature [5]. This case is the first of its kind in our country to our knowledge. The male predominance of African histoplasm has been reported in most series with a sex ratio between 2 and 3 [2, 5]. Our patient was immunocompetent. The recent African literature mentions very few cases of African histoplasmosis var duboisii associated with HIV infection [4]. Contamination is often done by yeasts found in the soil and materials contaminated by bird or bat droppings [1]. The reservoir of histoplasmosis *capsulatum var. duboisii* is considered to be in Africa because published cases have been reported in people living on this continent, especially in West and Central Africa [2,6].

### CLINICAL

Clinical presentation may be localized with isolated skin, bone, or lymph node infections or disseminated with multiple cutaneous lesions present all over the body, subcutaneous abscesses, enlarged lymph nodes, liver and spleen, and visceral organ enlargement [1,3]. The cutaneous manifestations are isolated or associated with type of nodules, papules, ulcers as in our case [7]. It can be subcutaneous swelling, taking the form of a cold abscess progressing to spontaneous fistulization, or ulcers, sometimes budding [7]. If neglected, the lesions may extend to give a large ulcer [4].

### PATHOLOGY

Rapid diagnosis of histoplasmosis in Africa is only currently possible using microscopy; antigen testing and polymerase chain reaction are not available in most of Africa [2]. Histopathological, the fungi accumulate within macrophages where they grow and bud, forming a granuloma made up of very large giant cells comprising fungi with surrounding inflammation and fibrosis [2]. Inflammatory granulomas are formed of epithelioid cells, lymphocytes, neutrophils and many giant cells, containing many small round or oval types of yeast. The yeast cell sizes of the duboisii variant are larger, measuring 10–15 μm compared with 2–5 μm for the capsulatum variant [8]. Yeasts are easily identified in tissue sections by virtue of time-honored histochemical stains, such as PAS or Grocott methenamine-silver. Differential diagnosis mainly includes *Cryptococcus* and *Penicillium* species: the lack of a mucicarminophilic halo excludes *Cryptococcus*, whereas *Penicillium* yeasts divide by intracellular septation, not by narrow budding [8].

### TREATMENT AND EVOLUTION

Treatment of Africa histoplasmosis involves surgical clearance of isolated cutaneous lesions and antifungal drug therapy for deep lesions and disseminated disease [9]. Our patient, being immunocompetent, followed a course of oral itraconazole for 12 months. However, in patients with AIDS, the recommended treatment includes an intensive phase of 3 months with amphotericin B replaced by itraconazole (400 mg/d) for severe forms or itraconazole alone (600 mg/d) for 3 days, then 400 mg/d) for mild forms [9]. Prolonged maintenance therapy with itraconazole (200 mg or 400 mg/d) is recommended in immunocompetent because relapses may be observed several years after the first episode. Similarly, prolonged follow-up is mandatory for every patient with histoplasmosis due to variety duboisii [10].
